# Wound of the Trachea—Occlusion of the Larynx—Aerial Fistula

**Published:** 1831

**Authors:** 


### LIII.

### Wound of the Trachea—Occlusion of the Larynx—Aerial Fistula. By M. Renaud, (Journ. Hebdom.)

*Case.* J. M. Leblanc, suspected of assisting in the fabrication of false money, was induced to quit his home, and seclude himself in another part of the country. In the mean time, it seems that he was condemned to death for contumacy. "Pendant son absence, il est condamné à mort par contumace." Three years afterwards, (March 1821,) then 28 years of age, being in a field, he perceived three gendarmes approaching him; and conceiving that they were in pursuit of him, he instantly determined on suicide. He always carried a bistoury with him; and seizing the instrument with his right hand, he plunged it into that part of his throat corresponding with the space between the cricoid cartilage and trachea. The point of the instrument being directed upwards, it entered the larynx, and made its way out again, being directed from one side to the other. A profuse hæmorrhage ensued; and his answers to the gens-d'armes were not intelligible. They conveyed him to the nearest village, where he was unable to procure professional assistance for several hours. It was found necessary to introduce fluids into the stomach, for the purpose of alimentation, by means of a tube, and in twenty days the patient began to have some power of articulation. But, in proportion as the external wound healed, the difficulty of breathing increased; and in six weeks after the accident, the unhappy Leblanc, fearing the officers of justice, contrived to make his escape to a distant part of the country, where he look refuge with his brother. Then the terrible difficulty of breathing suggested to Leblanc the idea of re-opening the original wound, in hopes of either putting an end to his life or his sufferings. With this intention he took an opportunity of pushing a knife through the cicatrix, and thus giving a free vent to the respiration. In this auto-operation, Leblanc made an opening into the pharynx, but of small extent. His brother arriving in an hour, was terrified, and applied to the magistrate of the place, who procured a physician to examine into the state of the patient. He was conducted to the HÔTEL DIEU of Rheims, where an attempt was made to re-unite the wound; but the difficulty of breathing which ensued, caused them to abandon the attempt. The event was left to Nature, and in a fortnight the wound of the pharynx was healed. In proportion as the laryngeal wound healed, however, the dyspnœa increased, as on the former occasion; and to prevent suffocation, the patient himself constructed a tube of lead, two inches in length, and more than an inch in circumference, which he introduced, with some difficulty, but which gave him complete facility of breathing. He was obliged, of course, from time to time, to remove the tube, in order to clean it, and give issue to accumulated mucosities. In two months, he was completely well, with the exception of the inconvenience of the tube. And now the unhappy man was brought before a tribunal of justice, and was condemned to death. The severity of the sentence was, however, mitigated into perpetual labour. He was sent to work at a public construction in Toulon, where he arrived on the 11th September, 1822. There he worked till the month of August, 1825, when the leaden tube slipped into the trachea, and became impacted at the origin of the right bronchus. There it excited constant and violent fits of coughing. He was sent to the hospital, and the instrument was extracted by a surgical operation, no details of which are given. During the patient's stay in the hospital, M. Renaud ascertained the complete occlusion of the larynx, by various experiments; and yet the patient was able to articulate many words with very considerable distinctness. Many of the most distinguished medical men at Toulon corroborated these facts. They all became convinced that the articulation of sounds in Leblanc's case, was made in despite of the entire occlusion of the larynx. This man could speak so distinctly, as to be heard and understood at some distance. There were certain words and letters, however, which he could not pronounce, as, for example, the letters a, c, l, and especially o. When he attempted to speak, he opened his mouth wide, depressed the larynx, and then, by a violent effort, expelled what air he could, as if by the act of coughing. Leblanc became the subject of repeated attacks of bronchitis, which ended in phthisis, of which he died on the 28th July, 1828.

The dissection was made in the presence of the COUNCIL of HEALTH, and various officers of the hospital. The complete occlusion was satisfactorily proved, the obliteration of the passage being where the trachea joins the larynx. The problem remains to be solved how Leblanc could speak, under such circumstances. Our readers may remember the case of Mr. Price, of Portsmouth, who still breathes through a tube in the trachea. In his case there is a small aperture still for air, though not sufficient for respiration. His voice is almost extinct.

